# Feasibility and Efficacy of Magnetic Sphincter Augmentation for the Management of Gastroesophageal Reflux Disease Post-Sleeve Gastrectomy for Obesity

**DOI:** 10.1007/s11695-022-06381-6

**Published:** 2022-12-06

**Authors:** Leena Khaitan, Michael Hill, Michael Michel, Patrick Chiasson, Philip Woodworth, Reginald Bell, Ragui Sadek, Aaron Hoffman, Kari Loing, Paula Veldhuis, William Petraiuolo, Carlos Anciano

**Affiliations:** 1 Division of Thoracic & Foregut Surgery, Department of Cardiovascular Sciences, University Hospitals Cleveland Medical Center, Cleveland, OH 44106 USA; 2 Adirondack Surgical Group, Saranac Lake, NY 12983 USA; 3 Coastal Carolina Bariatric & Surgical Center, Summerville, SC 29485 USA; 4 Northwest Allied Bariatric & Foregut Surgery, Tucson, AZ 85741 USA; 5 Institute of Esophageal & Reflux Surgery, Englewood, CO 80113 USA; 6 Advanced Surgical and Bariatrics of New Jersey & Pennsylvania, Somerset, NJ 08873 USA; 7 Kaleida Health, Buffalo, NY 14203 USA; 8 Medical Affairs, Ethicon Inc, 4545 Creek Rd, Cincinnati, OH 45242 USA; 9 Division of Thoracic & Foregut Surgery, Department of Cardiovascular Sciences, East Carolina University, Greenville, NC 27834 USA

**Keywords:** Magnetic augmented sphincter, MSA, Laparoscopic sleeve gastrectomy, Weight loss surgery, Gastroesophageal reflex disease, GERD, LINX®

## Abstract

**Background:**

Patients with medically intractable GERD after laparoscopic sleeve gastrectomy (LSG) have limited surgical options. Fundoplication is difficult post-LSG. Roux-en-Y gastric bypass may be used as a conversion procedure but is more invasive with potential for serious complications. Magnetic sphincter augmentation (MSA) is a less invasive GERD treatment alternative. The objective of this study was to assess safety and efficacy outcomes of MSA after LSG.

**Methods:**

The primary outcome of this observational, multicenter, single-arm prospective study was the rate of serious device and/or procedure-related adverse events (AEs). The efficacy of the LINX device was measured comparing baseline to 12-month post-implant reductions in distal acid exposure, GERD-HRQL score, and average daily PPI usage.

**Results:**

Thirty subjects who underwent MSA implantation were followed 12 months post-implant. No unanticipated adverse device effects were observed. There were two adverse events deemed serious (dysphagia, pain, 6.7%) which resolved without sequelae. GERD-HRQL scores showed significant improvement (80.8%, *P* < 0.001), and reduction in daily PPI usage was seen (95.8%, *P* < 0.001). Forty-four percent of subjects demonstrated normalization or >  = 50% reduction of total distal acid exposure time (baseline 16.2%, 12 months 11%; *P* = 0.038).

**Conclusions:**

Post-LSG, MSA showed an overall improvement of GERD symptoms, and reduction in PPI use with explants within anticipated range along with improvement in distal esophageal acid exposure time.

**Graphical Abstract:**

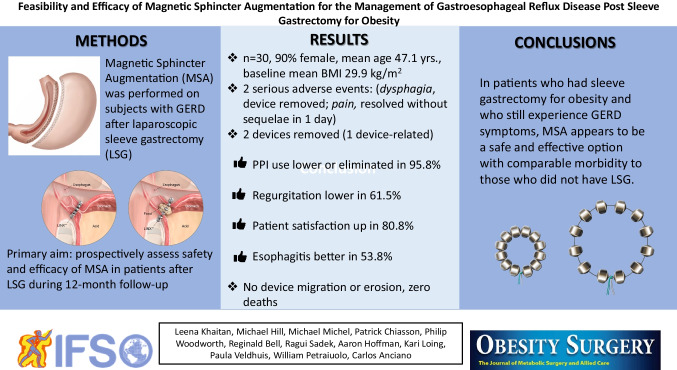

## Introduction

Globally, the prevalence of individuals with morbid obesity is rising with bariatric surgery being widely utilized as a surgical weight loss option [1]. Laparoscopic sleeve gastrectomy (LSG) is the most commonly performed restrictive weight loss surgical procedure [2, 3]. The majority of individuals with pre-existing gastroesophageal reflux disease (GERD) that undergo LSG will continue to experience GERD symptoms, and up to 26.7% of patients that did not have pre-existing GERD will develop new onset GERD symptoms [4, 5]. Unfortunately post-LSG, if weight loss, diet modification, anti-reflux medications, and lifestyle changes do not successfully mitigate symptoms, fundoplication, the traditional surgical method of treatment, is not an option for this subset of patients due to the resected fundus. The Roux-en-Y gastric bypass (RYGB) has been recommended as a conversion procedure for those who develop or continue to experience reflux after LSG, but it is invasive with potential for significant complications [6].

**Fig. 1:**
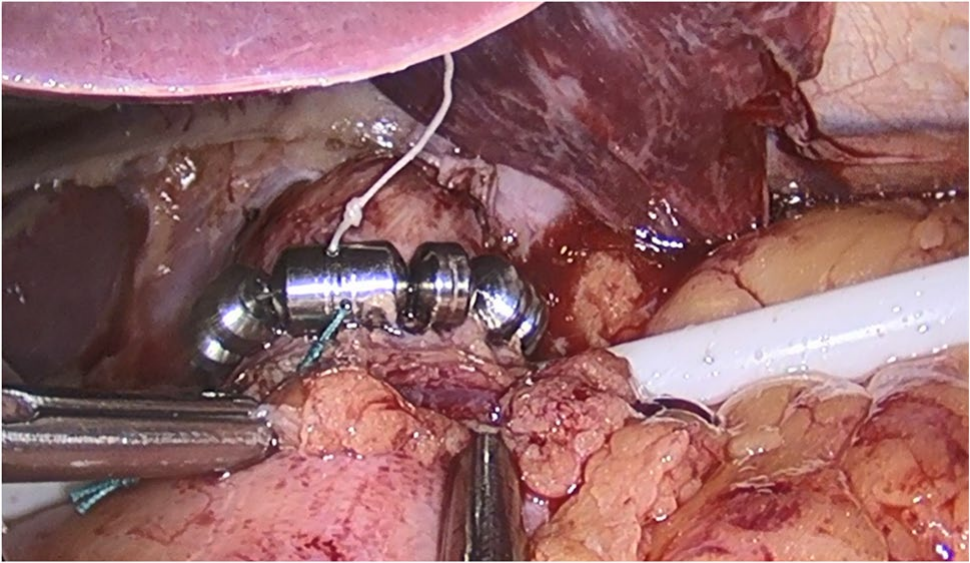
Implanted de novo LINX device

Magnetic sphincter augmentation (MSA), utilizing the LINX Reflux Management System (LINX), has been considered an alternative treatment option for patients experiencing GERD symptoms post-LSG [7–11]. LINX is a fundic-sparing anti-reflux device placed laparoscopically around the esophagus at the level of the lower esophageal sphincter (LES) (Fig. 1). It is currently indicated for placement in patients diagnosed with GERD as defined by abnormal pH. The device design and mechanism of action have been previously described in detail [12–14]. Briefly, the device consists of a series of titanium beads with a magnetic core connected with independent titanium wires to form an annular shape when implanted. The attractive force of the magnetic beads provides additional support to keep a weak LES closed. During swallowing, the magnetic beads slide away from each other on the wire “links” to allow esophageal distention as the bolus passes by. The principal aim of this Investigational Device Exemption (IDE) study was to evaluate the safety and efficacy of MSA in patients who had previously undergone LSG.

## Methods

### Study Design and Patient Population

This prospective, post-market, single-arm, multi-site observational study was designed to evaluate the safety and efficacy of MSA post-LSG (ClinicalTrials.gov identifier: NCT02429830) and was conducted from December 1, 2017, to June 8, 2021, at 12 US institutions. This study included 30 subjects presenting with GERD, who were screened, provided informed consent, and subsequently implanted with a MSA device. IRB approval was obtained at individual sites and the study conducted in accordance with the Helsinki Declaration, 21 CFR Parts 11, 50, 54, 56, 812, and local regulations.

Eligibility included surgical candidates ≥ 22 years of age, able to tolerate general anesthesia, and laparoscopic surgery who had undergone LSG for obesity at least 12 months prior to proposed implantation with documented symptoms of GERD persisting longer than 6 months requiring daily proton pump inhibitor (PPI) or other anti-reflux drug therapy. Further primary inclusion criteria conformed with the previous pivotal study and can be found on clintrials.gov. Exclusion criteria were restrictive to match the previous pivotal study. Primary criteria included body mass index (BMI) > 35; scleroderma; esophageal or gastric varices; history of Barrett’s esophagus; esophageal motility disorder; history of known esophageal stricture or gross esophageal anatomic; presence of Grade C or D esophagitis; and suspected or known allergy to titanium, nickel, stainless steel, or ferrous materials.

At baseline/screening, subjects underwent a work-up to determine eligibility for anti-reflux surgery which included an esophageal endoscopy, pH testing, manometry/motility, and a barium esophagram (Table 1).

**Table 1:** Assessments performed and data collected

Visit	Assessments/data collected
Screening/baseline	• Informed consent and inclusion/exclusion criteria assessed • Demographics (date of birth, gender, race) • Height and weight • GERD history: duration of PPI use, years with GERD, GERD new onset or pre-existing after LSG • GERD-HRQL (on/off PPIs) and Foregut Symptoms Questionnaire (off PPIs) • Baseline GERD-related medication use • Esophageal pH measurements (off PPIs) • Endoscopy • Manometry/motility • Barium esophagram • Motivation for surgery
Implant/discharge	• Surgery date • Surgery start and stop time • Implanted device size • Concomitant procedures (e.g., hiatal hernia repair, cholecystectomy) • Barium esophagram and upright Bi-Planar X-Rays (AP and Lateral) • Discharge date • Perioperative and device- and/or procedure-related adverse events
2-week	• Device- and/or procedure-related adverse events
3-month	• GERD-related medication usage within the last 30 days • GERD-HRQL (off PPIs) and Foregut Symptoms Questionnaire (off PPIs) • Device- and/or procedure-related adverse events
6-month	• GERD-related medication use within the last 30 days • GERD-HRQL (off PPIs) and Foregut Symptoms Questionnaire (off PPIs) • Device- and/or procedure-related adverse events
12-month	• Height and weight • GERD-HRQL (off PPIs) and Foregut Symptoms Questionnaire (off PPIs) • GERD-related medication usage within the last 30 days • Esophageal pH testing (off PPIs) • Manometry/motility • Endoscopy • Barium esophagram and upright Bi-Planar X-Rays • Device- and/or procedure-related adverse events

#### DEVICE AND IMPLANTATION

The LINX Reflux Management System (Torax Medical, Inc., Shoreview, MN, part of the Johnson & Johnson family of companies) received Food and Drug Administration (FDA) approval on March 22, 2012. Standard principles of re-operative interventions are followed with lysis of adhesions, crural dissection, and repair with preservation of peritoneal lining, hiatal hernia reduction with restoration of intrabdominal esophageal length by mediastinal mobilization, and preservation of vagal nerves. LINX system is placed in through window inside posterior vagal nerve and outside anterior one, sitting just above the laparoscopic GEJ and endoscopic Z-line after sizer tool used at same level. Post-sleeve gastroplasty anatomy does not change these intrinsic principles for device implantation [15].

#### SAFETY

was assessed by evaluating the rate of serious device- and/or procedure-related adverse events (AEs), perioperative complications, device malfunctions, device removals, and hospital re-admissions post-implant during a 12-month follow-up period. Esophageal anatomy and functionality were monitored via manometry and barium esophagram at 12 months post-implant allowing for identification of any potential abnormal or atypical findings. Esophageal integrity was assessed by endoscopy at the 12-month visit. Subjects who required explant were monitored for 3 months post-removal for AEs.

#### EFFICACY

was assessed by evaluating GERD control before and after device placement, based upon esophageal pH measurements, GERD-HRQL scores, and PPI use. Subjects served as their own controls. The change from baseline to follow-up was calculated and summarized as follows:

1. Esophageal acid exposure time (normalization for total distal ambulatory esophageal pH testing was defined as pH < 4 for < 4.5% of the time)
2.  ≥ 50% reduction in total GERD-HRQL score (off PPIs)
3.  ≥ 50% reduction in average daily PPI dosage

### Questionnaires

Two self-rating questionnaires, the validated Velanovich reflux severity symptom assessment (GERD-HRQL) and the Foregut Symptoms Questionnaire (FSQ) to assess regurgitation symptoms, were administered at baseline, and then at 3, 6, and 12 months [16].

### Statistical Plan and Additional Analyses

While no formal statistical hypotheses were pre-defined, summary statistics were used to display results of outcomes at each time point. For categorical parameters, this included the number and frequency; and for continuous parameters, the mean, median, standard deviation, range, and 95% confidence limits. For continuous efficacy parameters, the *P*-value for a one-sample, paired *t*-test was utilized to evaluate if there was statistical evidence of change from baseline is ≠ 0 is also given.

## Results

Of the fifty subjects who provided informed consent, 20 were not implanted (*n* = 18 failed to meet criteria; *n* = 2 met criteria but due to logistics were not enrolled) and considered screen failures. The remaining 30 were enrolled/implanted. The study population consisted of 90.0% female with a mean age of 47.1 years with an average baseline BMI of 29.9 kg/m^2^ (Table 2). The mean length of time between LSG and MSA implantation was 3.2 years. New onset GERD symptoms post-LSG surgery were seen in 30.0% of subjects. Hiatal hernia was observed after both endoscopy and esophagram with barium swallow in 63% of subjects at baseline (mean 2.2 cm). Subjects reported having GERD symptoms for an average of 11.6 years and taking PPIs for an average of 7.5 years prior to MSA implantation.

**Table 2:** Subject baseline demographics

Variable	Characterization	Number of subjects (%) ^[1]^ (*N* = 30)
Age at consent (years)	Mean (SD)	47.1 (12.1)
	Median (min, max)	43.0 (27.0, 73.0)
Gender	Male	3/30 (10.0%)
	Female	27/30 (90.0%)
Race	White or Caucasian	27/30 (90.0%)
	Black or African American	2/30 (6.7%)
	Other	1/30 (3.3%)
Ethnicity	Hispanic or Latino	2/30 (6.7%)
	Not Hispanic or Latino	28/30 (93.3%)
Height (cm)	Mean (SD)	164.4 (6.7)
	Median (min, max)	163.0 (155.0, 178.0)
Weight (kg)	Mean (SD)	80.8 (11.1)
	Median (min, max)	80.0 (57.0, 101.0)
Body mass index (kg/m^2^)	Mean (SD)	29.9 (3.2)
	Median (min, max)	30.5 (23.1, 34.9)

^[1^^]^All percentages are calculated using the number of subjects in the FAS as the denominator

The average surgical time for MSA implantation was 58.1 ± 23.3 min with most subjects being discharged the same day (0.7 nights ± 0.6). Ninety percent of subjects had concomitant crural or hiatus repair. Note that investigators were queried regarding combined crural reinforcement and hiatal hernia repair, so the authors are unable to distinguish between the two adjunct procedures. Crural “reinforcement” is expected post-dissection of the lower esophagus and essential in the context of this type of surgery for GERD. One perioperative complication occurred in a subject who experienced a pneumothorax during LSG staple line dissection off the pleura. An 8 Fr pigtail catheter was inserted intraoperatively for management and surgery resumed. The subject was discharged later the same day and experienced a full uncomplicated recovery. The distribution of device size was 15 beads (40.0%), followed by 16 beads (26.7%), 17 beads (23.3%), and 14 bead device (10.0%). The majority of LINX implant procedures were associated with concomitant surgical procedures (90.0%), most commonly hiatal hernia and/or crural repair (90%, aggregate calculation).

Two subjects required device removal at 17 and 121 days respectively. At the 3-, 6-, and 12-month visits, the subject compliance for follow-up was, 29/29, 28/28, and 27/28, respectively. One subject missed the 12-month visit and was determined lost to follow-up. No deaths were reported during the study period.

### Safety Results

A total of 21 device- and/or procedure-related adverse events occurred in 15 subjects of which 19 were anticipated. The two unanticipated AEs included one case of moderate intensity face tingling and pain, and one subject experienced mild intensity esophagitis. Both were deemed not serious (Table 3). Two AEs were deemed serious and occurred in different subjects both of which required hospitalization longer than 24 h. One patient experienced dysphagia, nausea, and vomiting which was deemed likely device-related and resulted in device removal. One subject experienced pain requiring prolongation of hospitalization.

**Table 3:** Adverse events

Adverse events term	Number of events	Number of subjects (%) ^[1]^ (*N* = 30)
Total	21	15/30 (50.0%)
Other ^[2]^	8	8/30 (26.7%)
Dysphagia	5	5/30 (16.7%)
Pain	3	3/30 (10.0%)
Nausea	2	2/30 (6.7%)
Diarrhea	1	1/30 (3.3%)
Esophageal spasm	1	1/30 (3.3%)
Pneumothorax	1	1/30 (3.3%)

^[1^^]^All percentages are calculated using the number of subjects in the FAS as the denominator

^[2^^]^Other:

Epigastric pain and bloating (*n* = 1)

• Esophagitis (*n* = 1)

• Face tingling and pain (*n* = 1)

• Foam pooling in throat (*n* = 1)

• Hypersensitivity to dermabond (*n* = 1)

• Pleural effusion (*n* = 1)

• Post-op atelectasis and pleural effusion (*n* = 1)

• Vomiting, pain in chest and nausea (*n* = 1)

During the study, 2 re-admissions occurred. One subject was re-admitted 3 days post-implant for shortness of breath and left chest/flank pain. A chest CT revealed bilateral pleural effusions with compressive atelectasis. The subject underwent a thoracentesis the next day resolving the effusion and was then discharged. This was followed by two additional Emergency Department visits for right leg swelling and left shoulder pain which did not require admission on either occasion. The other subject was re-admitted 8 days post-implant with nausea, vomiting, and dysphagia (as mentioned below). After IV hydration and prednisone, the subject was discharged 4 days later. Symptoms however did not resolve, and the subject was eventually explanted.

Two devices were safely explanted without complications. One subject was explanted (as noted above) on day 17 post-implant for dysphagia, and one 121 days post-implant with conversion of sleeve to gastric bypass due to the subject’s anatomy (dilated fundus) which caused dysphagia. Both subjects were followed 90 days post-explant and no AEs noted.

Manometry/motility testing showed no significant abnormal or atypical findings between baseline and 12-month follow-up. Results from barium esophagram evaluations showed normal swallowing function at baseline 100.0%, post-implant prior to discharge 93.3%, and 12 months 96.2%. There were no reported device malfunctions, device migrations, or device erosions at 12 months.

### Efficacy Results

A ≥ 50% reduction in total distal acid exposure was achieved in 11/24 subjects, and 12/27 of subjects experienced normalization or ≥ 50% reduction in total acid exposure. pH normalization was attained in 6/27 subjects. The total percent time in reflux with a ≥ 50% reduction in total distal acid exposure decreased from 16.2% at baseline to 11.0% at 12 months (*P* = 0.038) (Table 4). At 12 months post-LINX implant, 80.8% of subjects reported at least a 50% reduction in total GERD-HRQL scores when compared with baseline (*P* < 0.001) (Table 5).

**Table 4:** Changes in pH (baseline to 12 months)

Variable	Category/statistic	Number of subjects (%) ^[1]^
Baseline: total % time	Mean (SD)	16.2 (6.4)
	Median (min, max)	15.5 (6.5, 28.1)
	Number (missing)	30/30 (0)
	95% CI of mean	13.8, 18.6
12 months: total % time	Mean (SD)	11.0 (11.0)
	Median (min, max)	7.5 (0.6, 43.3)
	Number (missing)	24/27 (3)
	95% CI of mean	6.3, 15.6
Change from baseline to 12 months: total % time	Mean (SD)	− 5.0 (11.1)
	Median (min, max)	− 6.0 (− 22.6, 31.8)
	Number (missing)	24/27 (3)
	95% CI of mean	− 9.7, − 0.3
	*p*-val (dif to 0, *t*-test)	0.038
12 months: subjects with pH normalization (< = 4.5%)	Yes	6/27 (22.2%)
	No	21/27 (77.8%)
12 months: subjects with > = 50% reduction in total distal acid exposure	Number (missing)	24/27 (3)
	Yes	11/24 (45.8%)
	No	13/24 (54.2%)
12 months: subjects with normalization or > = 50% reduction	Yes	12/27 (44.4%)
	No	15/27 (55.6%)

^[1^^]^Denominator and percentages are based on subjects with non-missing data

**Table 5:** GERD-HRQL scores off PPIs (baseline to 12 months)

Variable	Category/statistic	Total
Baseline: GERD-HRQL total score	Mean (SD)	35.6 (9.7)
	Median (min, max)	36.0 (19.0, 50.0)
	Number	30
	95% CI of mean	32.0, 39.2
12 months: GERD-HRQL total score	Mean (SD)	8.1 (11.3)
	Median (min, max)	2.5 (0.0, 36.0)
	Number	26
	95% CI of mean	3.6, 12.7
Change from baseline to 12 months: GERD-HRQL total score	Mean (SD)	− 26.0 (12.7)
	Median (min, max)	− 26.5 (− 50.0, − 2.0)
	Number	26
	95% CI of mean	− 31.1, − 20.9
	*p*-val (dif to 0, *t*-test)	< .001
Subjects with at least 50% reduction on GERD-HRQL ^[1]^	Missing [2]	4
	Yes	21/26 (80.8%)
	No	5/26 (19.2%)

^[1]^Denominator and percentages are based on subjects completed baseline and 12-month follow-up. % Successful is defined as number of subjects meeting the success criterion of at least 50% reduction on GERD-HRQL at 12 months follow-up compared to baseline off PPI

Overall, the percentage of subjects who reported being dissatisfied with their GERD symptoms went from 96.7% (29/30) at baseline to 23.1% (6/26) at 12 months. Note that at baseline one subject responded “neutral” regarding symptomology both on and off PPIs but reported a 15 to 20-year history of severe heartburn, mild regurgitation, and occasional difficulty swallowing while presenting with a DeMeester score of 63.8 and grade B esophagitis. At 12 months, 95.8% subjects experienced a ≥ 50% reduction in daily use of PPI medication (*P* < 0.001) and 84.6% of subjects reported discontinuing all PPIs. The average daily DeMeester score at baseline was 54.1 and at 12 months decreased to 35.1 (*P* = 0.005). The number of daily reflux episodes decreased from a mean of 68.6 to 55.0, with the longest episodes per day decreasing from 45.1 to 29.6 min, respectively (Table 6).

**Table 6:** Changes in DeMeester score components utilizing daily averages

Variable	Category/statistic	Baseline	12 M follow-up	Change from baseline to 12 M follow-up
Total % time	Mean (SD)	16.0 (6.3)	11.4 (10.7)	− 4.7 (10.8)
	Median (min, max)	15.3 (6.5, 28.0)	7.8 (0.6, 43.6)	− 5.9 (− 16.6, 32.1)
	Number (missing)	29 (0)	24 (0)	24 (0)
Upright % time	Mean (SD)	14.8 (7.9)	10.9 (10.0)	− 4.2 (11.8)
	Median (min, max)	14.0 (2.5, 34.7)	9.3 (0.8, 43.5)	− 6.4 (− 20.8, 34.7)
	Number (missing)	29 (0)	24 (0)	24 (0)
Supine % time	Mean (SD)	15.3 (10.7)	7.2 (10.9)	− 7.9 (10.4)
	Median (min, max)	14.9 (0.0, 37.2)	2.5 (0.0, 42.2)	− 9.2 (− 28.3, 13.0)
	Number (missing)	29 (0)	23 (1)	23 (1)
# of reflux episodes	Mean (SD)	68.6 (33.9)	55.0 (70.6)	− 13.5 (62.3)
	Median (min, max)	62.0 (11.0, 159.0)	29.0 (5.5, 317.0)	− 21.0 (− 89.5, 177.0)
	Number (missing)	29 (0)	24 (0)	24 (0)
# of reflux episodes > 5 min	Mean (SD)	8.7 (4.1)	5.9 (4.9)	− 3.1 (5.1)
	Median (min, max)	7.8 (3.0, 18.0)	4.8 (0.0, 18.5)	− 3.4 (− 13.5, 11.5)
	Number (missing)	29 (0)	24 (0)	24 (0)
Longest episode (min)	Mean (SD)	41.5 (27.0)	29.6 (19.0)	− 14.0 (30.1)
	Median (min, max)	35.5 (13.5, 136.5)	25.8 (3.0, 69.0)	− 11.8 (− 97.5, 33.5)
	Number (missing)	29 (0)	24 (0)	24 (0)
DeMeester score	Mean (SD)	54.1 (21.6)	35.1 (33.3)	− 20.2 (32.2)
	Median (min, max)	52.7 (20.4, 96.7)	24.3 (2.4, 131.0)	− 25.0 (− 59.7, 80.7)
	Number (missing)	29 (0)	24 (0)	24 (0)
	*p*-val (dif to 0, *t*-test)			0.005
# Subjects with normal DeMeester score based on daily average (< = 14.72)	Yes	0 (0%)	4 (16.7%)	21 (87.5%)
	No	29 (100.0%)	20 (83.3%)	3 (12.5%)

Subjects reported a decrease in frequency of regurgitation symptoms post-implant. At baseline, regurgitation occurred 26.4 times/week compared to 3-, 6-, and 12-month visits, where frequency was 7.9, 1.8, and 4.6 times/week, respectively. Severe regurgitation was reported in 36.7% of subjects at baseline and improved to 3.4% of subjects at 3 months with no reported cases at 6 or 12 months. Moderate regurgitation was reported in 43.3% subjects at baseline and at 3-, 6-, and 12-month visits was 3.4%, 7.7%, and 7.7% of subjects, respectively. Mild regurgitation was reported in 16.7% of subjects at baseline, 27.6% at 3 months, 23.1% at 6 months, and 30.8% at 12 months. The number of subjects having no regurgitation increased from baseline (3.3%) to 65.5%, 69.2%, and 61.5% at follow-up (3, 6, and 12 months). Additionally, 40.0% of subjects reported one or more extra esophageal symptoms at baseline which decreased to 6.9% at 3 months, 11.5% at 6 months, and 15.4% at 12 months. Based on esophagogastroduodenoscopy testing at baseline, 63.3% of subjects experienced esophagitis which decreased to 11.5% at 12 months post-implant. Grade A esophagitis was reported in 7/30 subjects at baseline while at 12 months, 2/26 had Grade A esophagitis. No Grade B esophagitis was noted at 12 months comparing to the 40.0% at baseline though one subject experienced worsening from Grade B (baseline) to Grade C (12 months). Of note, this subject had a 2-cm hiatal hernia at baseline which was repaired during the MSA procedure. An apparent re-herniation occurred sometime post-procedure which measured 4 cm at 12 months with a Hill Grade of III.

## Discussion

Sleeve gastrectomy, in which approximately 75–85% of stomach is removed, is routinely utilized as a weight loss surgical option after which patients often report increased GERD symptoms or de novo ones [17]. Traditionally, the preferred first line of treatment for GERD after lifestyle modification is anti-reflux medications but for many patients, this treatment is ineffective in entirely reversing the symptoms [18–20]. In those who have had weight loss surgery, the fundus that is traditionally used for the LES augmentation has been removed rendering such intervention impossible. Thus, MSA has been employed though patients with prior gastric surgery, such as LSG, have not been enrolled in MSA clinical trials to date. Therefore, we aimed to assess safety and efficacy of MSA for patients seeking an alternative to acid suppression for GERD after LSG.

Since FDA approval for uncomplicated GERD, MSA’s safety and effectiveness have been well established in over 160 peer-reviewed publications to date. Our post-market study was conducted under an IDE with the intent of removing the precaution in labeling concerning use of MSA in patients having undergone LSG. The same inclusion/exclusion criteria as the initial pivotal Pre-Market Approval trial were employed in this study. All subjects had surgical alteration of gastric and gastroesophageal junction anatomy with the performance of a gastric sleeve resection. Recent evidence shows that physiologic alterations in sleeve gastrectomy patients are observable through manometry as compared to patients without altered anatomy. Jaruvongvanich, et al. performed a meta-analysis on esophageal pathophysiologic changes after bariatric surgery [21]. The review included 27 studies with 612 sleeve gastrectomy (SG) and 470 Roux-en-Y gastric bypass subjects. After SG, LES pressure and esophageal body amplitude decreased, and the risk of ineffective esophageal motility increased. For the 10 sleeve studies, pooled LES pressure decreased an average of 3.6 mmHg. Total and recumbent acid exposure times were increased. Balla et al. conducted a systematic literature review, and also noted manometric and pH monitoring changes after LSG [22]. A total of 21 studies with manometric data for 402 patients were included. A decrease of the LES resting pressure after surgery was observed in 6 out of 8 studies, while worsening of the DeMeester score was observed in eight of 10 studies. No meta-analysis was not performed due to the heterogeneity of data.

Patient selection remains vital as one of the issues leading to reflux after sleeve gastrectomy can be attributed to sleeve morphology. If patients are noted to have a narrowing in the sleeve, a stenosis at the incisura, or dilation of the proximal sleeve above the fundus, then these morphologic issues may be the cause of the postoperative reflux [23, 24]. In these patients, simply addressing the LES may not be adequate to resolve the reflux and a revision of the dilated portion of fundus to create a more tubular shape may be required. However, the high intraluminal pressure nature of this tubular anatomy and watershed blood supply associates these revisions with higher leak rates. Therefore, many patients with these configurations may benefit more from a conversion to gastric bypass.

In this present study, patients generally tolerated the procedure well with significant improvement in symptoms revealing that MSA can be done safely in the post-LSG population with morbidity similar to that seen in those who had a primary MSA without a sleeve [25, 26]. The intervention was found to be effective in the management of GERD, most notably for improvement of regurgitation, esophagitis, and reduction in PPI use. While LSG is routinely performed, it has been identified as a particularly refluxogenic procedure with de novo rates as from 8.4 to 26.7% reported after the procedure [4, 5]. Thus far, the primary tool surgeons have used to help these patients is conversion of the sleeve to a gastric bypass procedure. The gastric bypass has been effective as it is a drainage procedure where the acid burden in the gastric pouch is reduced, and for those with a BMI over 35, it has led to enhanced weight loss [27].

It is well documented that the co-existence of a hiatal hernia (HH) can be a significant factor in causing increased esophageal acid exposure. The potential for a large unrepaired HH was considered a confounding factor for determining device efficacy in the initial research. Subjects were therefore excluded from this study if they had a hiatal hernia > 3 cm based on alignment with the pivotal protocol and initial product labeling [15]. The presence of a hiatal hernia (via endoscopy) was reported in 19 of 30 subjects at baseline with a mean size of 2.2 cm. Almost all patients had a concomitant hiatal hernia repair or cruroplasty. Subjects underwent both endoscopy and esophagram with barium swallow at baseline. Both of these diagnostics have limitations in terms of capturing evidence of hiatal hernias and measuring size if present, is somewhat subjective. As such, the results of the presence of 63% of hiatal hernias in this subject population may have been higher in actuality.

When complication rates are compared, those having MSA de novo versus those after prior sleeve gastrectomy showed no difference in serious adverse events [8, 9]. When minor events were reviewed, even the dysphagia seen in this IDE trial resolved quickly (see table below). As reported by Asti et. al., explantation is necessary on occasion primarily due to unresolved GERD symptoms or dysphagia with numbers similar to those observed in this study [28]. Explantation rates were similar in both groups and consistent with those in the general population who had MSA [29]. Migration and erosion were not seen at 12 months after MSA in LSG patients.

When compared to the pivotal PMA trial, MSA after sleeve gastrectomy showed similar safety. In the pivotal trial, 9 serious AEs were reported in 6 subjects (*n* = 96) and 2 (*n* = 30) in the current study. Similarly, related AEs were 162 (*n* = 100) and 21 (*n* = 30) in the pivotal and current study, respectively. Of the 100 devices implanted in the pivotal study, 4 were removed while 2 were removed (out of 30) in the RELIEF study.

The effectiveness of MSA in the current study group was similar to that seen in the pivotal trial with regard to HRQL score improvement (pivotal 92% [92/100]; RELIEF 80.8% [21/26]), ≥ 50% reduction of daily PPI use (pivotal 93.0% [93/100]; RELIEF 95.8% [23/24]), improving esophagitis (pivotal 35.1% [34/97]; RELIEF 53.8% [14/26]), and eliminating regurgitation (pivotal 57.9% [55/95]; RELIEF 61.5% [16/26]). Interestingly, those in the post-sleeve gastrectomy group (35.1% [24]) did have higher DeMeester scores at 12 months post-procedure than those in the pivotal trial (18.7% [95]). Patient satisfaction, however, still remained high as evidenced by the improved GERD-HRQL scores.

While both DeMeester scores and pH were significantly improved but not normalized during the 12-month study period, subjects generally reported an improved satisfaction off PPIs at 12 months (76.9%) compared with the pivotal study (85%) [30]. Initially post-implant patient satisfaction at 3 and 6 months was slightly higher than at 12 months. This non-significant decrease is a variable to follow long-term, where postoperative progressive scarring, body weight changes, diet liberalization, patient expectations, and other factors may play a role. All LA grade B esophagitis receded, and only 2 patients showed remnant Grade A features. One subject experienced worsening esophagitis from Grade B to Grade C at 12 months. Collectively, esophagitis and extraesophageal symptoms improved significantly. Most importantly, two parameters which significantly affect quality of life, PPI use and regurgitation, showed significant improvement from their findings pre-MSA. One limitation of this study is the small subject number who were observed for a relatively short observation period (i.e., 12 months). Although the risk of device erosion is low, the median time to explant is 26 months with most occurring between 1 and 4 years after placement [31]. Additionally, non-bariatric literature suggests that more than 80% of explants (rate < 10%) occurred 1–2 years post-implant [32]. This study was constrained by the inclusion/exclusion criteria of the pivotal study, and thus, there is an element of selection bias in that all cases had successful weight loss (BMI < 35), with no (37%) or small (< 3 cm) hiatal hernias, and no Grade C or D esophagitis. Thus, the study population did not fully represent the post-sleeve GERD population. In addition, this technology is not intended to treat all GERD after sleeve as the causality is multifactorial. This study provides a GERD solution for a specific group of patients with GERD after sleeve as outlined with the inclusion criteria [15].

## Conclusion

Based on the 12-month results of this IDE trial, which specially evaluated MSA in a subject population who had achieved weight loss post-LSG, PPI use is significantly lower and even eliminated in most subjects. Regurgitation is also significantly reduced resulting in improved quality of life. Thus, MSA appears to be safe and effective with comparable morbidity in the treatment of GERD in patients who previously underwent sleeve gastrectomy.

## Data Availability

The data that support the findings of this study are available but restrictions apply to the availability of these data which is not publicly available. Data are however available from the authors upon reasonable request.
